# ‘Only to reconcile with it’. The coping experience amongst middle‐aged and older cancer survivors: A qualitative study

**DOI:** 10.1111/hex.14048

**Published:** 2024-04-12

**Authors:** Yi He, Wei Zhao, An Duan, Hong Xiao, Xuemei Zhou, Qiqi Zhuo

**Affiliations:** ^1^ Department of Cancer Center The Second Affiliated Hospital of Chongqing Medical University Chongqing China

**Keywords:** cancer, cancer survivors, coping, middle‐aged, older, qualitative research

## Abstract

**Background:**

Cancer threat is relevant to age, and the threat of a foreshortened life coupled with a lengthy treatment process negatively affects middle‐aged and older adults. Understanding the coping throughout the cancer experience in middle‐aged and older cancer survivors will help develop supportive care to promote their physiological and psychological coping effects.

**Objectives:**

To explore the cancer coping experiences of middle‐aged adults aged 40–59 and older adults over 60.

**Design:**

A descriptive phenomenological study was employed.

**Methods:**

Face‐to‐face, in‐depth, semistructured interviews were conducted with 22 oncology patients in a tertiary university hospital aged 40 or above from August to October 2023. The interview data were analyzed using thematic analysis procedures.

**Results:**

Five themes and 13 subthemes were formed through analysis: acceptance of cancer (considering cancer as chronic, believing in fate and attributing cancer to karma); having different information needs (desired to be truthfully informed, information‐seeking behaviour, information avoidance behaviour); getting families involved (developing dependent behaviours, feeling emotional support, family members suffering worse); striving to maintain positive psychological state (positive thinking, seeking peer support) and negative experience (undesirable, low self‐esteem).

**Conclusion:**

Our study reveals that cancer survivors' attitudes towards having cancer have changed from a death sentence to a more positive perception of a chronic disease. Supportive programmes for developing coping strategies should consider the cultural traditions and religious beliefs, different information needs, involvement of family and promoting a positive psychological state while avoiding negative factors.

**Patient or Public Contribution:**

Participants with experience of coping with cancer were involved in the semistructured interview.

## 1 BACKGROUND

Age is the greatest risk factor for developing cancer, and the majority of cancer cases occur in people over the age of 40.^1^ The aging population and advances in early detection and treatment technologies have extended their survival, resulting in an unprecedented growth in the number of middle‐aged and older cancer survivors.^1^, ^2^ However, it has been widely reported that middle‐aged and older cancer survivors lack professional support in coping with stressful situations during the cancer journey, including diagnosis, lengthy treatments such as surgery, radiotherapy, chemotherapy, hormone therapy and survival after treatment.^3^, ^4^ As many of them have limited experience in coping with stressors, the stressors may be overwhelming for them and are not conducive to better adaptation to the disease and improvement in quality of life; this in turn, causes adverse physical symptoms (e.g., vomiting, nausea, taste changes, sleep disturbances, difficulty concentrating, hypertension, fatigue, dyspnoea and pain) and psychological suffering (e.g., fear, anxiety and depression).^3^, ^4^, ^5^, ^6^ Therefore, to optimize outcomes for middle‐aged and older cancer survivors, providing healthcare support to improve coping effectiveness is indispensable in physical and psychological care.

Understanding how middle‐aged and older cancer survivors cope with cancer‐related stressors is fundamental to the establishment of a more supportive milieu during the disease journey.^7^ It has been reported that coping is an individual's cognitive and behaviour attempts to deal with stressful situations to meet various survival demands.^7^ Appropriate coping strategies are a beneficial factor in promoting posttraumatic growth^8^; it also implies a significant positive association with resilience, effective control of the patient's behaviour and emotions, higher quality of life, and better adaptation to the disease process.^3^ For instance, some cancer survivors believe that their cancer experience was God's will, fate or karma and that using religious coping can enhance clinician self‐care and equanimity.^9^, ^10^ Searching for health information is also considered an essential facet in improving coping with illness and treatment adverse effects.^11^ Furthermore, emotion‐based coping, seeking social support, cognitive restructuring, self‐blame, blaming others, fatalistic, avoidance and negation, and so forth, are also reported to play important roles in patients' coping with illness.^10^, ^11^, ^12^, ^13^, ^14^ However, most of these coping strategies were reported in quantitative studies, including all ages, and derived from the broad theoretical dimensions, failing to capture the pivotal experiences in developing these positive or negative coping strategies in middle‐aged and older cancer survivors.

Therefore, using a qualitative methodology is the ideal way to delve deeper into the actual experience of coping with cancer, the coping processes and the meaning of these coping strategies.^7^ Several qualitative studies have explored the coping strategies adopted by cancer patients, but the population of interest is adolescents and young adults, and few have explored the coping strategies of middle‐aged and older adults. For example, Wu et al.^12^ conducted open‐ended interviews to explore the coping experiences of adolescents with cancer receiving chemotherapy and found that losing confidence and rebuilding hope were their essential coping strategies. Bradford et al.^15^ found in their qualitative study that seeking support is an important coping strategy for adolescents and young adults aged 15–29. While middle‐aged and old adults differ significantly from adolescents and young adults in terms of physical, physiological, cognitive and behaviour characteristics.^16^ Therefore, conducting a qualitative study to understand how middle‐aged and older adults cope with cancer is essential to develop tailored intervention procedures to promote a better response. In this study, we conducted a qualitative study to explore how middle‐aged and older adults cope with cancer.

## 2 METHODS

### 2.1 Study design

We conducted a qualitative study employing the descriptive phenomenological method to acquire a profound insight into the reasons, processes and strategies employed by cancer survivors to cope with their condition. The narrative account method, which explores personal knowledge and experience about coping with cancer, is frequently employed to reveal the meaning of these experiences.^17^ Face‐to‐face semistructured interviews were organized to gather qualitative data.

### 2.2 Setting and sample

The study was conducted at a tertiary care hospital located in Chongqing Province, China. Purposive sampling was adopted to recruit participants with diverse characteristics, including varying ages, genders, ethnicities, literacy levels, disease diagnoses, disease duration, personality traits, and so forth. Cancer survivors were eligible to participate if they: 1—had been diagnosed with cancer for at least 1 year; 2—diagnosis informed; 3—ability to speak Mandarin; 4—age greater than or equal to 40 years; 5—no history of mental illness and 6—with a stable condition. A time limit of at least 1 year was selected to ensure that participants had sufficient exposure to the disease, enabling them to describe their personal experiences of coping with cancer. Potential participants were identified and approached for participation by nurses working in the hospital. Accordingly, nurses who were familiar with potential participants contacted them, explained the study's aim, and invited them to participate. All approached potential participants were willing to participate. No one dropped out of the study.

### 2.3 Data collection

Interviews were undertaken between April and September 2023. Face‐to‐face individual semistructured interviews were conducted by the corresponding author, a female nurse with a master's degree and experience in qualitative research but unfamiliar with participants. All the participants were interviewed individually in the department's clinical trials communication room or locations most comfortable for the participants. A first version of the interview guide was formulated based on theories related to coping and previous qualitative studies on cancer survivors' coping experiences.^12^, ^15^, ^18^ Experts in the field, including two medical oncologists, two nurses and two psycho‐oncologists, were then invited to check whether essential components to coping with the disease were missing. Pilot interviews were performed separately with three cancer survivors who met the inclusion criteria to adapt the interview guide and evaluate the interview process. The final semistructured interview guides included questions 1–3: Could you kindly describe your experience with diagnosis, treatment and posttreatment survivorship? Questions 4‐5: What cognitive and behavioural efforts did you employ when facing cancer? Questions 6—8: Did you encounter any difficulties in your illness journey? if so, what were they, and how did you cope with them? The interviewer posed one question at a time, following the interview outline and allowing participants 1–2 min for reflection or inquiry. For instance, can you describe your experience after being diagnosed? Can you describe your experience during treatment? All participants demonstrated comprehension of the interview questions and provided individual responses. All interviews were audio recorded. Simultaneously, the participants' key expressions, actions and emotions were recorded in notes during each interview. Interviews were conducted until data saturation was reached, evidenced by the fact that no new categories or themes had been identified.^19^ Data saturation was reached after the 20th participant was interviewed, while two additional participants were included to ensure no new themes emerged. Only one participant was interviewed twice because the interview was interrupted by a family visit.

### 2.4 Data analysis

Descriptive statistics were used to analyze demographic characteristics data. Participant recordings were transcribed verbatim in Chinese within 24 h by one author (H. X.) and afterward revisited by another author (XM. Z.) to verify that transcribing had been done correctly. Any unclear information was repeatedly required during the interview process, and consequently, the transcripts were not returned to participants for comments. Thematic analysis was employed to analyze the qualitative data.^20^ When listening to and reflecting on participants' coping experiences, the researcher shed all preconceptions, perceptions and prior knowledge related to the phenomenon under investigation. After all data were collected, two authors (W. Z., A. D.) with experience in qualitative research repeated reading the transcripts and field notes to become acquainted with the data; then used line‐by‐line analysis to generate initial codes. Similar codes were aggregated together and sublimated into themes. Any discrepancies were subsequently discussed until a consensus was reached between these two authors. After reaching a consensus, the obtained themes and representative quotations were translated from Chinese into English by a bilingual translator, and then another bilingual translator performed back‐translates to ensure that meaning was retained. Because the patient was discharged when the analysis was completed, the findings were not returned to the patient for confirmation.

### 2.5 Ethical considerations

We followed the Consolidated Criteria for Reporting Qualitative Research&amp;#x000A0;to report this study.^21^ Written informed consent, including the study's aims and procedures, the freedom to withdraw and a small number of sociodemographic questions, was provided to all voluntarily participating participants. The interview was scheduled after participants filled out written informed consent. To protect privacy and confidentiality, participant's names were replaced by numbers, and no personal data will be revealed at any time. There is no compensation for participating in this study.

## 3 RESULTS

A total of 22 cancer survivors aged 41–71 years volunteered to participate in the semistructured interviews, including 10 males and 12 females. The majority of participants were of Han ethnicity (95.5%). Educational levels ranged from illiteracy to university degrees. 40.9% were diagnosed with lung cancer, and the remaining participants were diagnosed with 11 types of cancer, including cervical, breast, pancreatic and others. The length of time to diagnosis ranged from 1 to 14 years. The demographic characteristics of the participants are presented in Table 1. Interviews ranged in duration between 28 and 143 min, with a mean duration of 68 min.

**Table 1:** Participant characteristics.

Characteristics	Participants, *N* (%)	Mean/SD (range)
Age (years)		53.4/9.5 (41–71)
Gender		
Male	10 (45.5)	
Female	12 (54.5)	
Ethnicity		
Han	21 (95.5)	
Yao	1 (4.5)	
Educational		
Junior high school or less	10 (45.5)	
High school	5 (22.7)	
College/university	7 (31.8)	
Diagnosis		
Lung cancer	9 (40.9)	
Cervical cancer	2 (9.1)	
Nasopharyngeal carcinoma	1 (4.5)	
Thymic carcinoma	1 (4.5)	
Ovarian cancer	1 (4.5)	
Liver cancer	1 (4.5)	
Cholangiocarcinoma	1 (4.5)	
Brain neoplasms	1 (4.5)	
Breast cancer	2 (9.1)	
Sigmoid neoplasms	1 (4.5)	
Pancreatic cancer	1 (4.5)	
Oesophageal cancer	1 (4.5)	
Years since diagnosis		
		2.9/3.0 (1–14)

Five themes for coping experiences derived from the data: (1) acceptance of cancer; (2) having different information needs; (3) getting families involved; (4) striving to maintain a positive psychological state and (5) negative experience.

### 3.1 Theme 1: Acceptance of cancer

When talking about their experiences regarding coping with cancer, no participants judged, avoided or denied the illness. Participants with all diagnoses acknowledged the reality of the disease and accepted the losses it brings while striving to live with cancer. Our participants adopted certain cognitive reframing strategies to acceptance the illness, which included three categories: (a) considering cancer as chronic; (b) believing in fate and (c) attributing cancer to karma.

#### 3.1.1 Category 1: Considering cancer as chronic

Most participants considered cancer to be a common chronic disease. They noted that most people with cancer can survive for extended periods and have no need to worry and fear about having cancer because, like hypertension, diabetes, or other chronic diseases, cancer can be controlled or even prolonged with long‐term treatment.

Patients with hypertension and diabetes do not live longer without long‐term medication. I thought the treatment was much more advanced than it used to be and could prolong my life for a long time … If all aspects of immunity and medication can control the disease, we can coexist with cancer. It seems that cancer is defined as a chronic disease, right? (Participant 13, aged 41)

#### 3.1.2 Category 2: Believing in fate

Some participants attributed cancer to fate, demonstrating that there is nothing one can do to control the disease, its severity, or the length of survival; as if it is all predetermined and cannot be artificially changed. These people thought that the best way to live with cancer is to give up useless resistance, cooperate with treatment and accept the inevitable.

How long I can live in this life is predestined, just as getting cancer is predestined and cannot be changed. All I can do is cooperate with the treatment, medication, tests, and follow‐ups, and just let nature take its course as to when I will die. (Participant 7, aged 68)

#### 3.1.3 Category 3: Attributing cancer to karma

Few participants interpreted that every cause has a corresponding effect, describing cancer as a punishment for sins committed by their family members or themselves in this life or a past life. With each experience of the painful that cancer brings, they clear a little bit of sins. Attributing cancer as karma helps them to accept death with equanimity.

We all have karmic sins, which may come from this life or previous life or my elders, and I have to pay them off. Every time I experience suffering, I repay some of it, and my karmic sins decrease. I feel that if I have paid it off in this way, it is deserved, just like being punished for sins. (Participant 5, aged 48)

### 3.2 Theme 2: Having different information needs

Participants expressed their different information‐seeking experiences and needs, which included three categories: (a) desired to be truthfully informed; (b) information‐seeking behaviour and (c) information avoidance behaviour. Participants desired to be truthfully informed about their diagnosis and prognosis, but had different needs for further information about the disease, which manifested in different information‐seeking behaviours. Some participants constantly seek additional information about diseases through various means, but some participants tend to avoid inputting more information related to cancer.

#### 3.2.1 Category 1: Desired to be truthfully informed

All participants described their desire to be provided complete and truthful information about diagnosis and prognosis, but doctors and family members often suppress the fact of the disease, resulting in an inability to get the correct information about their condition, make the right medical decisions and plan better for the rest of their lives.

Why give patients unrealistic hope? We need to know if treatment is still necessary and how effective it can be, but no one will tell us the truth … you can only trust 40% of what doctors say, because 60% of what they say is from their own perspective, and they have to give you hope. (Participant 6, aged 51)

#### 3.2.2 Category 2: Information‐seeking behaviour

Some participants said that they felt fearful after being diagnosed because having a diagnosis of cancer signalled a huge health threat and death was imminent, but that access to health information could help them to know how to cope better with the disease, thus reducing the fear of not knowing what to do. As a result, they were accustomed to constantly seeking more detailed information about aetiology, treatments, side effects, medications, and so forth, from various sources.

I cannot ignore it. As they say goes, ‘a long illness makes a doctor’, I have come to know all aspects of my illness quite well through consulting doctors, reading reports and searching online. knowing this has helped me not to panic … I think it is definitely better to know a little more so that you can shoot the arrow on target. (Participant 3, aged 55)

#### 3.2.3 Category 3: Information avoidance behaviour

However, some participants mentioned that ‘too much information’ about cancer was overwhelming and would put an unbearable physical and psychological burden on them and that avoiding information about the disease would keep them in a good physical and psychological state.

When I was diagnosed with cancer, I never bothered to search for any disease‐related information, and I told my family not to either. Knowing too much can cause me a lot of negative emotions, and the more I know, the more upset and anxious I become. I don't want to add psychological burden to myself. (Participant 5, aged 48)

### 3.3 Theme 3: Getting families involved

All participants acknowledged that the psychological and physiological effects of cancer were overwhelming for them both at diagnosis and over time, but that seeking and accepting support from family members could help them cope well at all stages. There were three categories: (a) developing dependent behaviours; (b) feeling emotional support and (c) family members suffering worse.

#### 3.3.1 Category 1: Developing dependent behaviours

Most participants reported that they benefited from engaging in dependency behaviour. Since they were ill, their family members had taken care of them and arranged everything for them since their illness, and they were used to depending on them in every aspect.

Since I've been ill, I don't care about anything. It's my family that takes care of me, registers me, pays for me, and arranges everything for me … my family also helps me with consultations and reminds me to take my medication. I don't know what I would do without them. (Participant 20, aged 54)

#### 3.3.2 Category 2: Feeling emotional support

Some participants felt that their family helped them cope with the disease by caring, encouragement, warm greetings, comfort, spending time together, and so forth, which gave them strength and helped them to navigate numerous difficult journeys and served as an impetus for their continued forward motion.

After I became ill, my family took care of me more carefully. I've experienced several relapses and metastases. My husband, parents, brother, sister, including children, are the ones who have sustained me through every difficult journey. It feels like they were always there for me, telling me not to think or worry too much. (Participant 15, aged 42)

#### 3.3.3 Category 3: Family members suffering worse

Some participants pointed out that their family members experienced more negative emotions than they did, which could be attributed to the fact that families worry about their loved ones, experience more challenges, and take on more responsibilities on their behalf, such as caring for the sick and coping with the economic difficulties.

When I told them the news, it was like a bolt from the blue. My sister cried all the time and my brothers were shocked too. I often turn around to comfort them, they are more anxious than I am … my wife had to manage all the household matters and now she is the one who holds our family together. (Participant 16, aged 59)

### 3.4 Theme 4: Striving to maintain a positive psychological state

Participants emphasized that they had tried to maintain a positive psychological state, as a positive psychological state is more important than treatment and positively associated with treatment outcome and even disease survival. The qualitative data resulted in two categories of maintaining a positive psychological state: (a) positive thinking; and (b) seeking peer support.

#### 3.4.1 Category 1: Positive thinking

Some participants stated that rather than being consumed by the perpetual fear of cancer, it was more beneficial to approach it in a positive light. Compared to accidents, at least cancer did not immediately claim one's life, and there was still time to complete unfinished tasks.

Life is about mindset, which accounts for 60%–80%. The better the mindset, the better the treatment effect … Compared to dying suddenly in an accident, I still have time to do what I haven't completed yet. (Participant 11, aged 65)

#### 3.4.2 Category 2: Seeking peer support

Some participants preferred talking to and hearing from patients who had experienced the disease and recovered well. They said that peer cancer patients have similar circumstances, could understand their situation, share common topics and, most importantly, give hope for treatment.

I once met an old adult who was 80 years old. He reassured me and said that having cancer was nothing. He said he had rectal cancer at the age of 52, and apart from having to wear a fecal bag on his belly, there was nothing else … which gave me a lot of encouragement. I said you lived 38 years with a fecal bag; I will live 39 years. (Participant 17, aged 61)

### 3.5 Theme 5: Negative experience

Throughout their experience with cancer, participants talked about some negative experiences that hindered the effectiveness of coping, including two categories: (a) undesirable; and (b) low self‐esteem.

#### 3.5.1 Category 1: Undesirable

A few participants mentioned that people around them deliberately stayed away from them after they had cancer because they thought cancer meant death and bad luck.

People who know you have cancer are scared of you and stay away from you for fear that you will infect them or bring them bad luck … My sister called me half a month before her birthday this year and asked me not to come to her birthday. (Participant 8, aged 68)

#### 3.5.2 Category 2: Low self‐esteem

A few participants reported that a series of side effects during the treatment process led to changes in appearance and physical weakness, making them distinct from healthy individuals and leading to low self‐esteem.

There's always a little bit of lack of confidence and low self‐esteem. Because we look sick on the outside when we come out of the hospital. Since I've been sick for over a year, I don't feel like a human being, so I definitely don't feel confident anymore. (Participant 19, aged 49)

## 4 DISCUSSION

This qualitative study explored the experiences amongst middle‐aged and older adults with cancer in relation to coping with cancer‐related stressors, which has been under‐reported. We identified five themes in the narrated experiences of coping with illness: (1) acceptance of cancer, (2) having different information needs, (3) getting families involved, (4) striving to maintain positive psychological state and (5) negative experience. Data revealed that middle‐aged and older patients with cancer had adopted positive cognitive and behavioural change strategies, but there are still some negative coping experiences during their cancer trajectory. The results seem to be important in developing healthcare support to help middle‐aged and older adults with cancer cope better during the cancer trajectory.

In our study, acceptance of the diagnosis, symptoms, limited life expectancy, and losses related to the illness are considered important coping strategies for middle‐aged and older cancer survivors. As in other studies, older cancer survivors are more likely to accept cancer and, as a result, report lower anxiety, depression, and suicide than younger adult cancer survivors.^22^, ^23^ Different from younger adults who have more negative beliefs about cancer, middle‐aged and older adults have an awareness that cancer is an ongoing chronic condition.^24^, ^25^ For this age group, fear may be temporary; moving towards a state of living with cancer as a chronic illness leads to adaption, reduction of fear, overcoming illness and planning for the future.^26^, ^27^ Furthermore, under the influence of traditional philosophies and religious beliefs, including ‘zhong sheng an si’, ‘spiritual immorality and the afterlife’, ‘life and death unified’, ‘fate’ and ‘cause and effect’, our participants attributed cancer to fate or karma, considering this approach important for improving physiological and psychological well‐being.^28^, ^29^ Gurm et al.^10^ also reported that middle‐aged and older cancer survivors formed a strong belief in cancer as fate or karma, and acknowledging this in helped them acceptance of their cancer and reframed it in a hopeful way. These results highlight the positive role of living with cancer as a chronic illness and incorporating traditional cultural and religious beliefs in disease coping.

Different information needs amongst middle‐aged and older cancer survivors have been identified when coping with cancer. Although the majority of family members opted to conceal unpleasant information to prevent their ill parents from deteriorating or even hastening their death.^30^ All participants in our study want full disclosure of cancer diagnosis and prognosis. Furber et al.^31^ also highlight the positive role of truthfully informed for middle‐aged and older cancer survivors in reducing anxiety and depression, making informed decisions, improving compliance and adherence to treatment, and better planning for a limited life. Furthermore, participants in our study demonstrated different information‐seeking behaviours when confronted with disease‐threat information. Agustina et al.^27^ similarly reported that almost a quarter of middle‐aged and older adults avoid searching for more cancer‐related information. The result might be explained by the ‘Blunting Hypothesis’, which suggests that individuals were categorized into monitoring information‐seeking styles (monitors) and blunting information‐seeking styles (blunters) with respect to cancer information.^32^ Monitors were dissatisfied with information provision and constantly sought cancer information from friends and family, health professionals, the Internet, printed materials and other sources, while blunters benefit from avoiding cancer‐related information; only information provision tailored to the individual's information‐seeking styles will make them better off psychologically, behaviourally and physiologically.^32^, ^33^, ^34^, ^35^ Hence, healthcare professionals should provide information about diagnosis and prognosis to people with cancer at the appropriate time and in the proper way, and deliver information tailored to the different information needs of individuals.

The involvement of family members in the experience of coping with the disease was highlighted in this study. As reported in earlier study that older cancer patients are expected to delegate their responsibilities more or less completely to their family members because they are unable or unwilling to do so; participants in our study expressed their dependence on spouses, parents, adult children or siblings for physical, emotional, social and financial support.^36^ While family members shoulder the full responsibility and caregiving tasks can prevent their ill loved one from feeling overwhelmed or in misery, it can also diminish patients' capabilities to cope with the long‐term effects of the disease, such as making informed choices and managing side effects, which in turn can lead to negative health outcomes.^37^, ^38^ Further, an increasing number of middle‐aged and older cancer survivors view family as their first source of emotional support to improve emotional well‐being throughout their cancer trajectory.^39^ Adolescent and young adult cancer survivors also believe that emotional support from family, including spending time together, listening attentively, providing encouragement and reassurance, making nice gestures and supporting choices, can help them cope with cancer diagnosis and treatment.^40^, ^41^ Besides, these participants described that the reason for uncertainty of the future, balancing care roles with job, family responsibilities, ill‐prepared and fear of the loss of a loved one led their family member to suffer distress, depression and sleep disturbances.^42^ In particular, family members of older cancer survivors have been reported to experience more emotional and physical strain.^43^ Healthcare professionals should consider patients and their families as a holistic care unit, providing physical and psychological care to patients and their family members to help families better involved in care, while encouraging patients to take a more active role in coping.

Positive psychological state is increasingly recognized as the most important facilitator of adjustment to the disease in middle‐aged and older cancer survivors. They were reported to have a positive view of the diagnosis, treatment and side effects of cancer, and to experience less shock, anger, sadness, depression and anxiety.^44^ These findings concur with Hoogland et al.^45^ who found that older cancer survivors demonstrate more positive psychological changes and higher levels of emotional resilience. Meanwhile, middle‐aged and older cancer survivors reported benefiting from peer support in the form of shared personal experiences or emotional relief from those in similar situations. Group or one‐to‐one peer mentoring provides them with information, social interaction, emotional relief, or practical help and is beneficial in maintaining their psychological wellbeing.^46^, ^47^ These findings highlight the importance of psychological support, such as helping patients with cancer promote and maintain positive thinking, as well as encouraging peer support to be more involved in cancer care.

However, there are still some negative experiences that may encumber effective coping. A few participants stated that their relatives or other social networks stayed away from them after knowing their diagnosis. This phenomenon has also been reported in young adult cancer survivors that their relatives, colleagues and friends become more distant or disappear completely during the cancer trajectory.^48^ The explanation for this phenomenon may be a lack of understanding of cancer amongst the general public or the cultural taboo of death.^48^, ^49^, ^50^ Additionally, negative impact on personal relationships, changes in physical appearance and fatigue greatly impacts cancer survivors' self‐esteem. This aligns with Skwirczyńska et al.^51^ showing that older prostate cancer patients are subject to many complications, which can significantly lower their self‐esteem and result in ineffective coping. This finding suggests that understanding and support from the cancer survivor's social surroundings is needed, while more interventions for higher self‐esteem need to be implemented.

### 4.1 Strengths and limitations

The experience of coping with cancer in middle‐aged and older adults is rarely explored in the literature. Our study provides a narrative voice about the perspective of middle‐aged and older cancer survivors on their experiences of coping with cancer. These findings add new information for health services and healthcare professionals in developing coping support programmes that can be of benefit to middle‐aged and older cancer survivors. There are still some limitations to consider. Our participants were recruited from a single geographic region, which reduces generalizability. However, purposive sampling allows us to enrol participants with diverse demographic characteristics and various cancer types, and in‐depth interviews allow for data saturation on key concepts, ensuring that the results are more generalizable. Additionally, we focus only on cancer survivors' perspective throughout the disease coping, whereas their informal caregivers and healthcare professionals may provide more comprehensive information about patients' coping experiences. Our approach of recruiting participants through nurses who knew them may have resulted in some participants not volunteering and should be used cautiously in future studies. Further research is needed to include the perspectives of family members and healthcare professionals on how people with cancer cope with the disease.

## 5 CONCLUSION AND IMPLICATIONS

The results show that middle‐aged and older adults with cancer undergo positive cognitive and behavioural modifications to better cope with the stressors of cancer, thereby maintaining physical and psychological well‐being throughout the cancer trajectory. Findings highlight the positive role of acceptance of cancer by recognizing it as a chronic disease and attributing it to fate or karma. Therefore, theory‐based psychological processes should be identified to fully capture the experience of living with cancer as a chronic illness to inform interventions to support patient adjustment; supportive care should consider and incorporate traditional cultural and religious practices to help patients cope effectively. More is not always better; on the one hand, being informed about the diagnosis and prognosis appropriately is a common demand; on the other hand, health information provision needs to be optimally adjusted to the information‐seeking styles of cancer survivors. There is no doubt that families provide vital support to partners coping with illness. Hence, home‐based support programs from healthcare professionals are needed to address caregivers' physical and psychological care needs to assist them in their caring role, but helping patients engage more actively in their self‐care is also essential for empowerment. The findings also call for positive thinking and peer support to be more involved in cancer care to maintain a positive psychological state. In addition, negative coping experiences are not conducive to coping with illness, so undesirable and low self‐esteem should be avoided.

## AUTHOR CONTRIBUTIONS

**Yi He**: Conceptualization; methodology; funding acquisition. **Wei Zhao**: Formal analysis; writing—review and editing. **An Duan**: Formal analysis; writing—review and editing. **Hong Xiao**: Data curation; writing—review and editing. **Xuemei Zhou**: Data curation; writing—review and editing. **Qiqi Zhuo**: Supervision; funding acquisition; project administration; writing—original draft; writing—review and editing.

## CONFLICT OF INTEREST STATEMENT

The authors declare no conflict of interest.

## ETHICS STATEMENT

Research ethics approval was obtained from the ethics committee of the host hospital (2023 ethical approval number 121).

## Supporting information

Supporting information.

## Data Availability

All data relevant to the study are included in the manuscript. Anonymized transcripts will be available on request from the corresponding author.
